# Modified-release gliclazide acutely improves recovery but causes undesirable blood glucose decrease after a resistance exercise session in healthy adults: a pilot study for a randomized clinical trial

**DOI:** 10.20945/2359-4292-2023-0381

**Published:** 2024-07-30

**Authors:** Jocelito B. Martins, Diego Zanella, Ramiro B. Nunes, Pilar S. Collado, Alexandre Machado Lehnen

**Affiliations:** 1 Instituto de Cardiologia do Rio Grande do Sul Fundação Universitária de Cardiologia Porto Alegre RS Brasil Instituto de Cardiologia do Rio Grande do Sul/Fundação Universitária de Cardiologia, Porto Alegre, RS, Brasil; 2 Departamento de Ciencias Biomédicas Universidad de León León España Departamento de Ciencias Biomédicas, Universidad de León, León, España; 3 Laboratório de Fisiologia do Exercício Centro Universitário da Serra Gaúcha Caxias do Sul RS Brasil Laboratório de Fisiologia do Exercício, Centro Universitário da Serra Gaúcha, Caxias do Sul, RS, Brasil; 4 Programa de Pós-graduação em Ciências da Reabilitação Universidade Federal de Ciências da Saúde de Porto Alegre Porto Alegre RS Brasil Programa de Pós-graduação em Ciências da Reabilitação, Universidade Federal de Ciências da Saúde de Porto Alegre, Porto Alegre, RS, Brasil

**Keywords:** Gliclazide, muscle recovery, resistance training, ergogenic effects, post-exercise recovery

## Abstract

**Objective:**

Sulfonylureas have been used to improve performance in strength sports. However, this hypothetical effect has not been proven. We examined the ergogenic acute effect of gliclazide on resistance training performance and muscle recovery.

**Subjects and methods:**

We conducted a double-blind, randomized, crossover pilot study with 10 healthy resistance-trained adults (29.3 ± 4.4 years), nonusers of anabolic steroids. The participants were randomized to two exercise sessions. In the first session, five participants received placebo and the other five received gliclazide modified release, both administered 8 hours before the session. Session two was performed in a crossover fashion a week later. The volume load was calculated as the maximum number of repetitions of four sets multiplied by load (65% 1-RM). Blood samples were collected before and after exercise, as well as 24 hours and 48 hours after exercise for measurement of creatine kinase (CK-MM) and lactate dehydrogenase (LDH) activity. Blood glucose was measured with a glucometer before, during, and after the exercise sessions.

**Results:**

Gliclazide did not enhance volume load for bench press (placebo: 2,698.0 ± 923.0 kg; gliclazide: 2,675.0 ± 1,088.0 kg; p = 0.073) or leg press (placebo: 10,866.0 ± 2,671.0 kg; gliclazide: 10,817.0 ± 2,888.0 kg; p = 0.135). However, CK-MM (-27.7%; p = 0.034) and LDH (-21.1%; p = 0.021) activities were decreased with gliclazide 48 hours after exercise. There was also a decrease in blood glucose in the gliclazide compared with the placebo session (p = 0.018).

**Conclusion:**

Gliclazide did not enhance performance in a single resistance training session, but promoted faster muscle recovery. The decrease in blood glucose post-exercise with gliclazide was an undesirable effect that could lead to long-term glucose metabolism disorders. Registered in ClinicalTrials.gov under number NCT04443777.

## INTRODUCTION

Sulfonylureas are insulin secretagogues used as a second-choice treatment in combination with lifestyle changes and metformin for type 2 diabetes management (1). Among sulfonylureas, gliclazide modified release (MR) is the preferred choice due to its lower rates of cardiovascular adverse events and hypoglycemia compared with other drugs in this class (2).

Gliclazide binding to sulfonylurea receptor-1 (SUR1) receptors blocks ATP-dependent potassium channels, stimulating membrane depolarization and opening voltage-dependent calcium channels in pancreatic beta cells; these effects lead to exocytosis of granules containing endogenous insulin and insulin release into circulation (3). The half-life of gliclazide is about 9 hours, ranging from 12 to 24 hours when administered at daily doses of 30-120 mg (3). These pharmacodynamic characteristics ensure stable gliclazide concentration in blood and reduce hypoglycemic events, making gliclazide safer for individuals without diabetes.

Gliclazide has potential ergogenic effects due to additional stimulus for endogenous insulin secretion, which is associated with increased muscle protein synthesis (chronic effect) (4) and greater energy availability (glucose) during exercise along with faster muscle glycogen replacement (acute effect) (5,6). This positive effect is due to greater glucose and amino acid uptake by myocytes, which acutely improves muscle glycogen replacement, repairs muscle tissue damage, and activates signaling pathways such as AKT-GLUT4 and AKT-mTOR-S6K (7) due to increased circulating insulin. Clinical use, (8) side effects, and associated risk of cardiovascular adverse events and hypoglycemia (9) with gliclazide in diabetes are well described in the literature. However, few studies have examined the potential effects of insulin secretagogues, especially gliclazide, on exercise performance and post-exercise recovery among athletes, non-athletes, and healthy individuals.

Despite insufficient data supporting the use of insulin secretagogues (such as gliclazide) as an ergogenic supplement and little knowledge about health risks associated with these substances, they have been used empirically to improve performance in professional athletes and non-athletes. Thus, the present study aimed to examine whether gliclazide has a potential ergogenic acute effect leading to enhanced exercise performance (considering volume load as the primary outcome) and post-exercise recovery (assessed by CK-MM and LDH activities, blood glucose level, and visual analogue scale [VAS] pain score as secondary outcomes) in healthy individuals undergoing a resistance training session. We hypothesized that the use of gliclazide favors resistance training performance in a single session and recovery from exercise-induced muscle damage.

## SUBJECTS AND METHODS

This randomized, double-blind, placebo-controlled, crossover pilot clinical trial is reported following the recommendations in CONSORT 2010 Statement: Extension to Randomised Pilot and Feasibility Trials (10). The study protocol was approved by the research ethics committee at *Instituto de Cardiologia do Rio Grande do Sul/Fundação Universitária de Cardiologia*, Porto Alegre, RS, Brazil (*Plataforma Brasil* CAAE 17434619.4.0000.5333 and approval number 3.771.137). The study was conducted according to the principles of the Declaration of Helsinki. The study is registered in ClinicalTrials.gov under number NCT04443777 (first registration on June 23, 2020).

### Study participants

Ten young men participated in the study after undergoing continuous resistance training for 2 years or more at a minimum frequency of 3 weekly sessions for the preceding 6 months. A convenience sample was used, as this was a pilot study that will serve as the basis for a sample calculation of a larger arm (randomized clinical trial). The exclusion criteria were self-reported acute or chronic use of anabolic androgenic steroids, anti-inflammatory drugs, beta-blockers, and exogenous insulin for the last 12 months; alcohol intake within 72 hours of the study intervention; bone and muscle injuries that hinder physical exercise; and non-adherence to dietary recommendations as instructed by the research team.

### Procedures

The study comprised 4 visits, with a 7-day interval between visits 3 and 4. On visit 1, the participants signed an informed consent form, underwent medical and body composition evaluation, and performed a one-repetition maximum (1-RM) test to become familiar with exercise sets. On visit 2, 1-RM tests were performed to determine the load for the experimental sessions. The experimental exercise sessions were held on visits 3 and 4; the participants were strongly advised not to exercise between sessions. Upon arrival at the study site (exercise laboratory), the participants were offered a pre-workout snack (1.2 g/kg carbohydrate and 0.12 g/kg protein). The experimental protocol was initiated 30 minutes later and started with bench press and leg press at a workload of 50% 1-RM. After a 2-minute rest, the resistance training session was started.

### Body composition evaluation

Body composition was assessed using the skinfold technique. A 7-site skinfold equation was used to estimate body density (11), and body fat was subsequently calculated using the Siri equation.

### One-repetition maximum tests

Upper limb maximum strength was assessed using a free-weight horizontal bench press, 3-inch model (TUTECH Equipamentos, Matelândia, PR, Brazil), and lower limb maximum strength was assessed using bilateral 45º leg press, 3-inch model (TUTECH Equipamentos). After a 5-minute warm-up on a cycle ergometer, the participants were asked to perform specific movements for the exercise test. The maximum load was determined in five attempts, with 5% load increments. The respective load was determined for a full range of motion. A 4-minute rest was allowed between attempts. An electronic metronome (KORG USA Inc., Melville, NY, USA) was used to control each repetition with 2 seconds for each contraction (concentric and eccentric) phase. Test-retest reliability coefficients (intraclass correlation coefficients [ICCs]) were > 0.97. The 1-RM tests were used to determine workloads for the study sessions (sulphonylurea [gliclazide] *versus* placebo).

### Exercise protocols

The exercise protocol consisted of four sets of bench press and leg press exercises at 65% of 1-RM with maximum repetitions until concentric failure. An electronic metronome (KORG USA Inc.) was used to control each repetition with 2 seconds for the concentric phase and 2 seconds for the eccentric phase. This intensity was set for the exercise because it is commonly used in recreational strength training to promote muscle hypertrophy (65%-85% of 1-RM) (12). Within this range of hypertrophy, we chose to set the lowest intensity (65%) at which exercise would be performed up to the concentric failure. Bench press exercises were followed by bilateral leg press exercises with no rest. A 2-minute rest was allowed after each set (bench press and bilateral leg press) of repetitions until concentric failure. Heart rate was measured using a Polar RS300 monitor (Polar Electro Oy, Kempele, Finland), and a VAS was applied for pain assessment (13) before each session, between each set of repetitions, and 24 and 48 hours after each session.

Resistance training volume was calculated for each type of exercise (bench press and leg press), and set and total training volume was calculated for each session as a product of exercise workload and number of sets and repetitions.

### Gliclazide and placebo administration

Gliclazide MR shows linear pharmacokinetic properties with increasing plasma levels up to 6 hours after administration, reaching a plateau after 12 hours (3). To take advantage of the window when plasma concentrations are higher, gliclazide and placebo were administered orally as matched capsules (same color, flavor, smell, and size) 8 hours before the beginning of each exercise session. Gliclazide 60 mg (Diamicron MR; Laboratórios Servier do Brasil Ltda., RJ, Brazil) or placebo (starch, sodium lauryl sulfate, and Aerosil) were randomly administered in a double-blind, crossover fashion 1 week apart.

Randomization of participants for placebo or gliclazide was performed with the use of a computer program (www.randomization.org) in 1:1 block with a coded numeric distribution (1-2). Allocation concealment was ensured; the random allocation of participants was kept in an inaccessible place and researchers did not have *a priori* knowledge of the intervention assignment to each participant. A researcher blinded to the study generated a numeric sequence for those participants meeting the inclusion criteria. All participants were blinded to group allocation (gliclazide or placebo) until the intervention day. The study evaluators were also blinded to the participants’ group allocation to minimize potential measurement biases.

The gliclazide dose was 50% of the maximum clinically recommended dose (120 mg) (14) and twice the minimum therapeutic dose (30 mg) (3,15). Of note, gliclazide has a half-life of 9-19 hours in healthy individuals (3,16). Evidence supports a 7-day washout period between visits (tests and experimental exercise sessions).

### Diet plan

Within 24 hours of both sessions, the participants were asked to follow a personalized diet plan prescribed by a skilled provider. The plan consisted of an estimated intake of total energy value of 18.5% from proteins (1.4 g/kg), 56% from carbohydrates (4.2 g/kg), and 25.5% from lipids (0.85 g/kg).

The participants were offered a custom snack (0.88 g/kg carbohydrate and 0.3 g/kg protein) (8) at the end of each exercise session to prevent potential hypoglycemic events and were instructed to follow their diet plan within 48 hours after each exercise session. They were additionally instructed to complete a food record for 24 hours before and 48 hours after the experimental sessions, enabling the researchers to verify adherence to the prescribed diet plan.

### Blood collection and analyses

Venous blood samples (5 mL) were obtained from the antecubital area after proper asepsis before, immediately after, and 24 and 48 hours after each session. After collection, blood samples were maintained at room temperature for 45 minutes and then centrifuged for 10 minutes at 2,300 x g, and serum was removed and frozen at -20 °C for later analysis. The activity of creatine kinase isoenzyme MM (CK-MM) and lactate dehydrogenase (LDH) in these serum samples were determined in duplicate using a colorimetric enzymatic device (Microplate Reader, Model TP-Reader NM, Thermo Plate, Robonik, India) according to the manufacturer’s guidelines for assay kits for CK-MM (Human CK-MM PicoKine ELISA Kit, EK1751, BosterBio, CA, USA) and LDH (Human Lactate Dehydrogenase B/LDH-B ELISA Kit AB183367, Abcam, USA). To eliminate inter-assay variance, all samples were analyzed within the same assay batch, and all intra-assay variances were ≤ 5.9%. Test-retest reliability coefficients (ICCs) were 0.82 for CK-MM and 0.84 for LDH. Data for serum CK-MM and LDH are presented in the International System of Units (µkat/L).

Capillary blood samples were collected from the participants’ fingertips for blood glucose determination using a glucometer (FreeStyle Optium; Abbott Laboratórios do Brasil, São Paulo, Brazil) before and immediately after each session (17). Data for blood glucose are presented in the International System of Units (mmoL/L).

Evidence shows that CK-MM returns to baseline levels 7 days after a strength training session (18,19). Similarly, after strenuous exercise, elevation of serum LDH activity is more pronounced within 3 days of exercise and LDH returns to baseline levels within 7 days (20).

### Statistical analyses

The data are presented as mean ± standard deviation values. The Shapiro-Wilk test was used to test the assumption of normality, and Levene’s test was used to assess the assumption of homogeneity of variance. For CK-MM and LDH activities, blood glucose levels, and VAS scores, repeated measures analysis of variance (ANOVA; Bonferroni *post hoc* tests) was used for comparisons of placebo *versus* glicazide over time, exercise sessions, and interaction between them. For comparison of volume load between exercise sessions, we performed Student’s *t* test (paired samples). Cohen’s effect size classification was also applied as non-relevant (0 to 0.19), small (0.20 to 0.49), medium (0.50 to 0.79), and large (above 0.80) (21). All data analyses were carried out using the statistical package SPSS, Version 23.0 (IBM Corp., Armonk, NY, USA) at a significance level of p < 0.05.

## RESULTS

**Figure 1:**
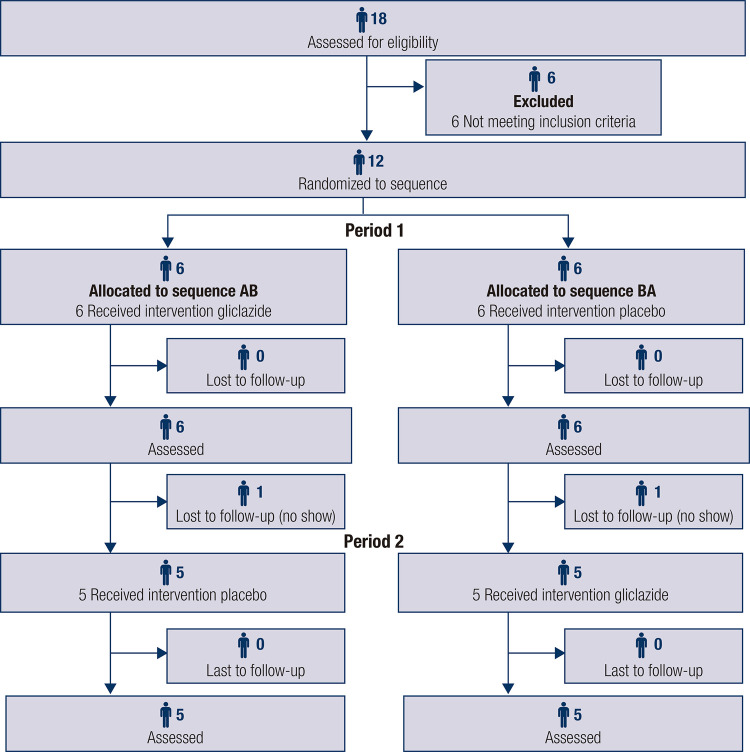
Flow chart of the study design. We conducted a randomized crossover clinical trial; 18 men were recruited to the study, but 6 did not meet the inclusion criteria. Thus, 12 participants were randomized to the study. After the first exercise session, 2 participants were excluded because they did not return for a subsequent session. Therefore, the final sample consisted of 10 participants.

Table 1 shows the general characteristics of the study sample. A total of 18 volunteers were initially recruited to participate in the study from November 2019 to July 2020 (recruitment and follow-up) at University of León. Of these, we excluded 6 participants who did not meet the study inclusion criteria and 2 who did not attend the second exercise session. Thus, our final sample comprised 10 participants who completed both exercise sessions of the study (Figure 1).

**Table 1:** General characteristics of the study sample (n = 10)

Age (years)	29.3 ± 4.42
Body mass (kg)	82.1 ± 11.0
Height (cm)	176.0 ± 3.68
Body fat (%)	13.16 ± 3.56
BMI (kg/m^2^)	26.48 ± 3.18
1-RM bench press (kg)	96.20 ± 18.77
1-RM bilateral leg press (kg)	308.50 ± 41.13

The data are presented as mean ± standard deviation values. Abbreviations: BMI, body mass index; 1-RM, one-repetition maximum test.

Table 2 shows the performance variables for each type of exercise. No difference was observed in the repetitions for each set (bench or leg press exercises), total exercise volume, and exercise volume per session. Also, there was no change in total training volume between gliclazide and placebo sessions (p = 0.123; Cohen’s effect size 0.02).

**Table 2:** Repetitions per set and total volume load

	Placebo session (n = 10)	Gliclazide session (n = 10)	P value	Cohen’s effect size
Bench press				
65% 1-RM (kg)	63.6 ± 13.4			
1st set – reps (n)	18.9 ± 4.2	18.5 ± 4.4	0.724	0.09
2nd set – reps (n)	10.6 ± 2.9	10.4 ± 3.7	0.764	0.06
3rd set – reps (n)	7.1 ± 3.0	6.8 ± 2.8	0.606	0.10
4th set – reps (n)	6.1 ± 2.7	6.1 ± 2.9	1.00	0.00
Total volume (kg)	2,698.0 ± 923.0	2,675.0 ± 1,088.0	0.823	0.02
Leg press				
65% 1-RM (kg)	201.2 ± 27.5			
1st set – reps (n)	19.4 ± 4.4	20.6 ± 5.0	0.373	0.25
2nd set – reps (n)	14.0 ± 2.9	13.5 ± 2.8	0.343	0.18
3rd set – reps (n)	10.9 ± 2.2	10.4 ± 3.2	0.544	0.18
4th set – reps (n)	9.6 ± 2.8	9.0 ± 3.3	0.394	0.10
Total volume (kg)	10,866.0 ± 2,671.0	10,817.0 ± 2,888.0	0.913	0.01
Total volume session (kg)	13,564.0 ± 3,594.0	13,492.0 ± 3,757.0	0.901	0.02

The total volume load was calculated as weight (65% 1-RM) X repetitions X set. Total exercise volume per session was the sum of exercise volume for each set. The data are presented as mean ± standard deviation values. Differences were tested using Student’s t test for paired samples for each type of exercise (p < 0.05). Abbreviations: reps, repetitions; 1-RM, one-repetition maximum test.

**Figure 2:**
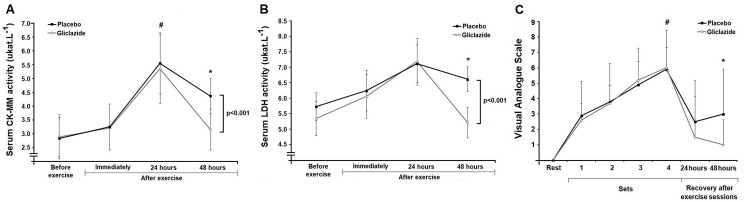
Muscle recovery pattern after resistance exercise sessions (n = 10). The data are presented as mean ± standard deviation values. The panels show comparisons of (A) creatine kinase MM (CK-MM) activity, (B) lactate dehydrogenase (LDH) activity, and (C) visual analogue scale (VAS) pain scores over time with placebo versus gliclazide. Differences were tested using repeated measures analysis of variance (ANOVA) with Bonferroni’s post hoc test. #P < 0.05 versus pre-session. *P < 0.05 versus placebo.

In both sessions, serum CK-MM activity increased 24 hours after exercise when compared with before and immediately after exercise (p value for interaction < 0.001), with no difference between gliclazide and placebo (Figure 2A). However, the gliclazide session had a lower serum CK-MM activity 48 hours after exercise compared with the placebo session (-27%; p = 0.034, Cohen’s effect size 1.75). Serum LDH activity varied over time and between sessions (p < 0.001), except for the time points immediately after exercise and in the subsequent 24 hours (Figure 2B). Indeed, serum LDH activity was lower 48 hours after exercise in the gliclazide session compared with the placebo session (-21%; p = 0.021; Cohen’s effect size 3.09). These data are shown in Table 3.

**Table 3:** Effects of gliclazide modified release (MR) on post-exercise recovery after resistance exercise sessions

	Placebo session (n = 10)	Gliclazide session (n = 10)	P value
CK-MM activity (µkat/L)			<0.001
Pre-session	2.83 ± 0.76	2.89 ± 0.80	0.513
Immediately post-session	3.25 ± 0.82	3.21 ± 0.86	0.404
24 hours after session	5.55 ± 1.10	5.35 ± 1.23	0.062
48 hours after session	4.36 ± 0.63	3.15 ± 0.74	<0.001
LDH activity (µkat/L)			<0.001
Pre-session	5.73 ± 0.44	5.34 ± 0.55	0.123
Immediately post-session	6.24 ± 0.66	6.06 ± 0.70	0.546
24 hours after session	7.11 ± 0.60	7.18 ± 0.75	0.788
48 hours after session	6.61 ± 0.40	5.21 ± 0.49	<0.001
Visual analogue scale (VAS) pain scores			0.123
Pre-session	0.00 ± 0.00	0.00 ± 0.00	1.000
Immediately post-session	5.90 ± 2.56	6.00 ± 1.33	0.899
24 hours after session	2.50 ± 2.68	1.50 ± 2.64	0.430
48 hours after session	3.00 ± 2.94	1.00 ± 1.63	0.017

The data are presented as mean ± standard deviation values. Differences were tested using repeated measures analysis of variance (ANOVA) with Bonferroni’s post hoc test. Abbreviations: CK-MM, creatine kinase isoenzyme MM; LDH, lactate dehydrogenase.

Subjective (VAS) pain scores are shown in Figure 2C. During the exercise sessions, subjective pain ratings were comparable between the gliclazide and placebo sessions (Figure 2C). During the recovery period, pain scores dropped by 58% in the placebo session and by 75% in the gliclazide session 24 hours after exercise and remained lower in the gliclazide session compared with placebo 48 hours after exercise (p = 0.032; Cohen’s effect size 0.84). These data are shown in Table 3.

**Figure 3:**
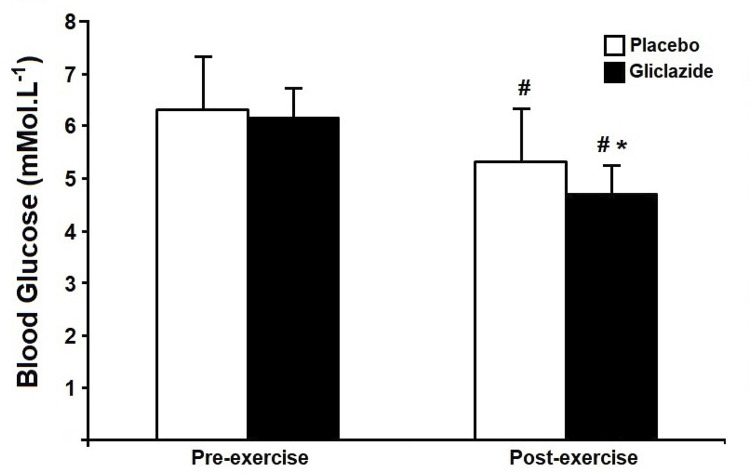
Blood glucose measurements before and after exercise in the gliclazide and placebo sessions (n = 10). Differences were tested by repeated measures analysis of variance (ANOVA) with Bonferroni post hoc tests. #P < 0.05 versus pre-session. *P < 0.05 versus placebo at the same time point.

Figure 3 shows blood glucose measurements before and after exercise sessions. There was a reduction over time (p value for time = 0.005) in both gliclazide (∆ = 1.45 mmoL/L; 24.32%; Cohen’s effect size 2.08) and placebo (∆ = 1.03 mmoL/L; 15.87%; Cohen’s effect size 0.98) sessions, but this reduction was more pronounced in the gliclazide compared with the placebo session (∆ = 0.61 mmoL/L; 11.46%; Cohen’s effect size 0.60).

## DISCUSSION

To the best of our knowledge, this is the first study examining potential ergogenic effects of a second-generation sulfonylurea (gliclazide MR 60 mg) on resistance training performance (assessed by total training volume load) and post-exercise recovery (CK-MM and LDH activities) after a single exercise session. We found that the acute use of gliclazide had no effect on training performance for bench press and leg press exercises. On the other hand, gliclazide was associated with more rapidly reduced markers of muscle damage during post-exercise recovery (48 hours after exercise), as assessed by lower serum enzyme activity of CK-MM and LDH and reduced pain scores when compared with placebo. Thus, we accept the hypothesis that acute use of gliclazide can improve post-exercise recovery, but we reject the hypothesis that it favors resistance training performance in a single session.

Two studies analyzing the chronic effects of insulin on muscle adaptation in response to exercise stimuli have shown that hyperinsulinemia potentiates muscle hypertrophy, amino acid uptake in myocytes, and protein synthesis (4,22). These effects are associated with long-standing training performance improvement. Since gliclazide increases endogenous insulin release, it is reasonable to assume it may induce, though to a lesser degree, the well-established effects of insulin on training performance. However, in the present pilot study including a small sample size, we found no effect of gliclazide on exercise performance in a single session. Some possible explanations for our results may be the dose of gliclazide MR (60 mg) used in the study, which was likely sufficient to stimulate additional insulin secretion (8). Patients with type 2 diabetes mellitus usually receive gliclazide MR at a dose of 60-120 mg (23), so we used the minimum recommended dose for diabetes management. Higher doses of gliclazide may be necessary to acutely enhance performance by increasing the relative amount of hormone released and binding to high-affinity receptors, along with greater translocation of glucose transporter type 4 (GLUT4) and insulin-sensitive glucose uptake. However, this hypothesis considers only a mechanistic effect. If we consider the effect from a health or performance perspective, there is an increased risk of hypoglycemia due to greater insulin release. It is well known that exercise inhibits insulin release (24) and increases glucagon secretion. Therefore, it is possible that, due to the interplay between the inhibition of insulin secretion during exercise and pharmacological stimulation of insulin secretion by gliclazide, the decrease in insulin secretion induced by exercise may have been more pronounced.

Another possible explanation for gliclazide being ineffective in inducing performance enhancement may be that the amount of glycogen stored in myocytes was greater than that demanded for the study exercise protocol (25). This scenario, together with the therapeutic window of gliclazide, may also have contributed to this finding. Pharmacokinetic studies of gliclazide MR show effects lasting up to 24 hours after administration (26), which can explain the apparent faster recovery with gliclazide compared with placebo found in the present study. To increase glycolysis in myocytes during exercise, an increase in pre-exercise glycogen would be necessary. Since pre-exercise glycogen stores were already fully loaded, there was no increased glucose entry into myocytes induced by gliclazide to promote glycogenesis. Therefore, irrespective of whether or not insulin secretion was increased due to gliclazide action, glycogen reserves in myocytes were at their maximum (27).

Gliclazide action improved recovery after exercise, probably by increasing glycogen replacement, as insulin concentrations are usually reduced after exercise due to an increase in glucagon (counterregulatory mechanisms) (24). Full glycogen stores may have limited the utilization of blood glucose as a substrate for glycolysis at the beginning of the session. Another possibility would be that if muscle glycogen stores were reduced, muscular glucose uptake would be higher in both settings (gliclazide and placebo) by translocation of GLUT4 independent of insulin mechanism (5). However, gliclazide may have been able to promote additional muscular glucose uptake, which was associated with a greater reduction in blood glucose after exercise in the gliclazide session, as shown in our results.

The post-exercise training period is marked by an alteration in the permeability of sarcolemmal membrane related to cellular recovery when there is an overflow of some cytoplasmic compounds such as CK-MM, lactate, and LDH. Thus, high blood levels of these markers can be an indirect predictor of muscle damage (28). We found peak serum CK-MM activity within 24 hours after exercise. This finding is consistent with the literature and allows us to infer that a single resistance training session was sufficiently vigorous to induce muscle damage (29). Post-exercise cell recovery involves the migration of leukocytes to the injury site, triggering an inflammatory response (26). Interestingly, gliclazide improved post-exercise recovery (CK-MM, LDH, and pain ratings) within 48 hours of exercise when compared with placebo.

Considering that gliclazide effects last up to 24 hours after its administration (23) and muscle glycogen stores are reduced following strenuous exercise, gliclazide seems to increase insulin secretion post-exercise and promote glycogenesis induction through insulin-mediated glucose uptake pathways (30), which would support the gliclazide effect of improving post-exercise recovery. Glycogen is restored in the muscles at a rate of 5%-7% per hour, and glycogen reserves are fully restored within 20 hours (25). This is extremely relevant since we found a faster recovery of exercise-induced muscle damage within 24-48 hours, mostly due to a reduction in serum LDH activity compared with placebo, assuming that intramuscular glucose metabolism was steady.

The time to plasma peak concentrations of gliclazide – within 6 hours after administration and lasting up to 12 hours – is also meaningful. Considering that, gliclazide levels remained high in the early recovery period (within 9 hours of administration), which potentiated insulin secretion and, consequently, glucose and amino acid uptake by myocytes, leading to enhanced muscle tissue repair. Together, these mechanisms accelerated the replenishment of muscle glycogen stores, which may explain lower serum activity of CK-MM and LDH within 48 hours of exercise in the gliclazide session. In addition, the lower late pain ratings seen in the gliclazide session compared with placebo also support the finding of improved recovery 48 hours after exercise.

Blood glucose kinetics varied throughout exercise sessions in both sessions. This finding can be explained by insulin-independent glucose metabolism associated with muscle contraction (31). Post-exercise blood glucose levels dropped by 28% (from 118 mg/dL pre-exercise to 85 mg/dL post-exercise) in the gliclazide session, a decrease that was significantly greater than the one observed in the placebo session (from 119 mg/dL pre-exercise to 99 mg/dL post-exercise). The decrease observed in the gliclazide session may have important clinical implications (post-exercise hypoglycemic events) and is a caution alert to potential side effects of gliclazide use by healthy volunteers, even at a low dose (60 mg) (9,32). A potential explanation for the post-exercise blood glucose drop is that increased insulin secretion due to pharmacological stimulation inhibits hepatic glycolysis, leading to a reduced amount of glucose released into the bloodstream. However, these are preliminary findings, and more supporting evidence is necessary.

Our study has some limitations worth noting. The main limitation is that we did not assess muscle function and performance post-exercise or insulin levels. Assessing muscle function as a dynamic force during recovery could provide further clarification of gliclazide effects on the repair of exercise-induced muscle damage. Insulin levels may have indicated whether the dose of gliclazide administered was sufficient to stimulate endogenous insulin secretion. In addition, measurements of inflammatory markers could have provided information about the extent of exercise-induced muscle damage and on how to improve exercise recovery. These are preliminary data from a pilot study of an arm of a randomized clinical trial. We are carrying out these assessments (muscle function during recovery, insulin levels, and inflammatory markers) to better understand the effects of gliclazide on exercise performance and recovery. Furthermore, the preliminary data in this study was obtained using a small sample size; thus, we cannot infer that gliclazide actually does not acutely improve performance in resistance exercise. The experimental design of this study (randomized with crossover intervention) allowed to minimize this potential bias. Controlling food intake also helped minimize these potential interferences.

In conclusion, for the sample of this pilot study, the use of gliclazide 60 mg did not improve resistance training performance in a single session. However, gliclazide seemed to have improved post-exercise recovery 24 to 48 hours after exercise (acute effect), as seen by lower serum activity of CK-MM and LDH and lower pain ratings. Post-exercise recovery improvement can be related to higher cellular glucose uptake, with consequent faster muscle glycogen store replacement and reestablishment of cellular homeostasis. This is a major finding that supports an ergogenic effect of gliclazide at the dose studied. Yet, it should be stressed that the use of gliclazide was also associated with an undesired decrease in blood glucose after exercise, in addition to the hypoglycemic effect of the exercise itself. Based on these preliminary results, the potential effects of gliclazide are in post-exercise recovery, but further examination in subsequent sessions is warranted. Hence, more scientific evidence is necessary to support the use of gliclazide for resistance training in healthy individuals.
